# A proposed framework for developing quality assessment tools

**DOI:** 10.1186/s13643-017-0604-6

**Published:** 2017-10-17

**Authors:** Penny Whiting, Robert Wolff, Susan Mallett, Iveta Simera, Jelena Savović

**Affiliations:** 1 NIHR CLAHRC West, University Hospitals Bristol NHS Foundation Trust, Bristol, UK; 2 School of Social and Community Medicine, University of Bristol, Bristol, UK; 3 Kleijnen Systematic Reviews Ltd., Escrick, York, UK; 4 Institute of Applied Health Research, University of Birmingham, Birmingham, UK; 5 National Institute for Health Research (NIHR) Birmingham Biomedical Research Centre, Birmingham, UK; 6 Centre for Tropical Medicine and Global Health, University of Oxford, Oxford, UK

**Keywords:** Risk of bias, Systematic reviews, Quality

## Abstract

**Background:**

Assessment of the quality of included studies is an essential component of any systematic review. A formal quality assessment is facilitated by using a structured tool. There are currently no guidelines available for researchers wanting to develop a new quality assessment tool.

**Methods:**

This paper provides a framework for developing quality assessment tools based on our experiences of developing a variety of quality assessment tools for studies of differing designs over the last 14 years. We have also drawn on experience from the work of the EQUATOR Network in producing guidance for developing reporting guidelines.

**Results:**

We do not recommend a single ‘best’ approach. Instead, we provide a general framework with suggestions as to how the different stages can be approached. Our proposed framework is based around three key stages: initial steps, tool development and dissemination.

**Conclusions:**

We recommend that anyone who would like to develop a new quality assessment tool follow the stages outlined in this paper. We hope that our proposed framework will increase the number of tools developed using robust methods.

## Background

Systematic reviews are generally considered to provide the most reliable form of evidence for decision makers [1]. A formal assessment of the quality of the included studies is an essential component of any systematic review [2, 3]. Quality can be considered to have three components—internal validity (risk of bias), external validity (applicability/variability) and reporting quality. The quality of included studies depends on them being sufficiently well designed and conducted to be able to provide reliable results [4]. Poor design, conduct or analysis can introduce bias or systematic error affecting study results and conclusions—this is also known as internal validity. External validity or the applicability of the study to the review question is also an important component of study quality. Reporting quality relates to how well the study is reported—it is difficult to assess other components of study quality if the study is not reported with the appropriate level of detail.

When conducting a systematic review, stronger conclusions can be derived from studies at low risk of bias, rather than when evidence is based on studies with serious methodological flaws. Formal quality assessment as part of a systematic review, therefore, provides an indication of the strength of the evidence on which conclusions are based and allows comparisons between studies based on risk of bias [3]. The GRADE system for rating the overall quality of the evidence included in a systematic review is recommended by many guidelines and systematic review organisations such as National Institute for Health and Care Excellence (NICE) and Cochrane. Risk of bias is a key component of this along with publication bias, imprecision, inconsistency, indirectness and magnitude of effect [5, 6].

A formal quality assessment is facilitated by using a structured tool. Although it is possible for reviewers to simply assess what they consider to be key components of quality, this may result in important sources of bias being omitted, inappropriate items included or too much emphasis being given to particular items guided by reviewers’ subjective opinions. In contrast, a structured tool provides a convenient standardised way to assess quality providing consistency across reviews. Robust tools are usually developed based on empirical evidence refined by expert consensus.

This paper provides a framework for developing quality assessment tools. We use the term ‘quality assessment tool’ to refer to any tool designed to target one or more aspects of the quality of a research study. This term can apply to any tool whether focused specifically on one aspect of study quality (usually risk of bias) or for broader tools covering additional aspects such as applicability/generalisability and reporting quality. We do not place any restrictions on the type of ‘tool’ to which this framework can be approach—it should be appropriate for a variety of different approaches such as checklists, domain-based approaches, tables or graphics or any other format that developers may want to consider. We do not recommend a single ‘best’ approach. Instead, we provide a general framework with suggestions on how the different stages can be approached. This is based on our experience of developing quality assessment tools for studies of differing designs over the last 14 years. These include QUADAS [7] and QUADAS-2 [8] for diagnostic accuracy studies, ROBIS [9] for systematic reviews, PROBAST [10] for prediction modelling studies, ROBINS-I [11] for non-randomised studies of interventions and the new version of the Cochrane risk of bias tool for randomised trials (RoB 2.0) [12]. We have also drawn on experience from the work of the EQUATOR Network in producing guidance for developing reporting guidelines [13].

## Methods

Over the years that we have been involved in the development of quality assessment tools and through involvement in different development processes, we noticed that the methods used to develop each tool could be mapped to a similar underlying process. The proposed framework evolved through discussion among the team, describing the steps involved in developing the different tools, and then grouping these into appropriate headings and stages.

## Results: Proposed framework

**Fig. 1:**
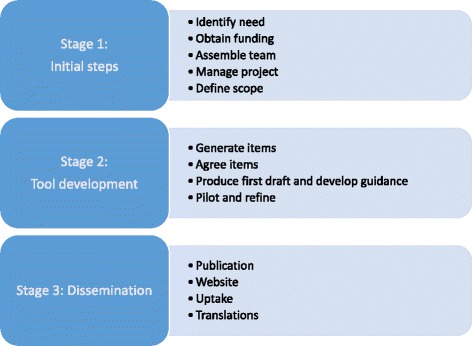
Overview of proposed framework

The Fig. 1 and Table 1 outline the proposed steps in our framework, grouped into three stages. The table also includes examples of how each step was approached for the tools that we have been involved in developing. Each step is discussed in detail below.

**Table 1:** Proposed steps to develop QA tools with examples drawn from existing tool development

Stage	QUADAS-2[8]	ROBIS [9]	PROBAST [10]	ROBINS-I [11]	RoB 2.0 [12]
Stage 1: initial steps					
1.1 Identify need	Update for QUADAS	New tool specifically for RoB in SR	New tool specifically for prediction models	New tool specifically for NRS of interventions	Update to Cochrane RoB tool
1.2 Obtain funding	Part of MRC methodology programme grant	MRC methodology project grant	No external funding	MIF grant from Cochrane and MRC methodology project grant	MRC hubs project grant
1.3 Assemble team	Larger group (*n* = 18)	Steering group (*n* = 10) + larger group (*n* = 19)	Larger group (*n* = 40)	Domain-based working groups and others (*n* = 35)	Domain-based working groups and others (*n* = 38)
1.4 Manage project	Steering group (*n* = 9)	Project group (*n* = 5)	Steering group (*n* = 9)	Steering group (*n* = 5)	Steering group (*n* = 8)
1.5 Define scope	Diagnostic test accuracy	Systematic reviews	Prediction modelling (prognostic and diagnostic)	Non-randomised studies of interventions	Randomised controlled trials
	Bias + applicability Domain based + signalling questions Signalling questions phrased so yes indicates low risk of bias	Bias + relevance Domain based + signalling questions Signalling questions phrased so yes indicates low risk of bias	Bias + applicability Domain based + signalling questions Signalling questions phrased so yes indicates low risk of bias	Bias Domain based + signalling questions	Bias Domain based + signalling questions + algorithm for domain level ratings
Stage 2: tool development					
2.1. Generate items	Starting point original QUADAS tool [7] Evidence review of sources of bias and variation	Review of existing QA tools + how QA handled in existing overviews Classification of MECIR guidance to identify sources of bias	Key publications on prognostic modelling studies Work on TRIPOD [21], CHARMS [22] and QUADAS-2 [8]	Survey of Cochrane review groups and face-to-face meeting. Domain-based working groups	Starting point draft tool based on original Cochrane RoB [23] tool. Evidence review of sources of bias.
2.2. Agree items	Face-to-face meeting	Face-to-face meeting	Web-based survey	Face-to-face meeting + domain-based working group	Face-to-face meeting + domain-based working group
2.3. Produce first draft	Steering group	Steering group	Steering group	Domain-based working groups + steering group	Domain-based working groups + steering group
2.4. Pilot and refine	Web-based survey + piloting in reviews	Web-based survey + piloting in reviews and by steering group members on key reviews	Web-based survey + piloting in reviews	Domain-based working groups, web survey, cognitive interviews, piloting by working groups and external reviewers on key papers, event to pilot version 1.0	Domain-based working groups + 3 day piloting event + additional piloting of refined version
Stage 3: Dissemination					
3.1 Publication	Single publication + guidance document as web supplement	Single publication + guidance document as web supplement	Primary publication + E&E document (in process)	Single publication + guidance document on website	Not yet published, guidance document on website
3.2 Website	www.quadas.org	www.robis-tool.info	Not yet available	www.riskofbias.info	www.riskofbias.info
3.3 Uptake	Recommended by multiple organisations including Cochrane, NICE and AHRQ,	Recommended by Cochrane, NICE and Estonia Health Insurance Fund	Recommended by Cochrane Prognosis Group	Not yet, only recently available	Not yet, only recently available
3.4 Translations	Italian and Japanese versions	Estonian and Portuguese versions in preparation	None to date	None to date	None to date

### Stage 1: initial steps

#### Identify the need for a new tool

The first step in developing a new quality assessment (QA) tool is to identify the need for a new tool: What is the rationale for developing the new tool? In their guidance on developing reporting guidelines, Moher et al. [13] stated that “developing a reporting guidelines is complex and time consuming, so a compelling rationale is needed”. The same applies to the development of QA tools. It may be that there is no existing QA tool for the specific study design of interest; a QA tool is available but not directly targeted to the specific context required (e.g. tools designed for clinical interventions may not be appropriate for public health interventions), existing tools might not be up to date, new evidence on particular sources of bias may have emerged that is not adequately addressed by existing tools, or new approaches to quality assessment mean that a new approach is needed. For example, QUADAS-2 and RoB 2.0 were developed as experience, anecdotal reports, and feedback suggested areas for improvement of the original QUADAS and Cochrane risk of bias tools [7]. ROBIS was developed as we felt there was no tool that specifically addressed risk of bias in systematic reviews [9].

It is important to consider whether a completely new tool is needed or whether it may be possible to modify or adapt an existing tool. If modifying an existing tool, then the original can act as a starting point, although in practice, the new tool may look very different from the original. Both QUADAS-2 [8] and the new Cochrane risk of bias tool used the original versions of these tools as a starting point [12].

#### Obtain funding for the tool development

There are costs involved in developing a new QA tool. These will vary depending on the approach taken but items that may need to be funded include researcher time, literature searching, travel and subsistence for attending meetings, face-to-face meetings, piloting the tool, online survey software, open access publication costs, website fees and conference attendance for dissemination. We have used different approaches to fund the development of quality assessment tools. QUADAS-2 [8] was funded by the UK Medical Research Council Methodology Programme as part of a larger project grant. ROBIS, [9] ROBINS-I [11] and Cochrane ROB 2.0 [12] were funded through smaller project-specific grants, and PROBAST [10] received no specific funding. Instead, the host institutions for each steering group member allowed them time to work on the project and covered travel and subsistence for regular steering group meetings and conference attendance. Freely available survey monkey software (www.surveymonkey.co.uk) was used to run an online Delphi process.

#### Assemble team

Assembling a team with the appropriate expertise is a key step in developing a quality assessment tool. As tool development usually relies on expert consensus, it is essential that the team includes people with an appropriate range of expertise. This generally includes methodologists with expertise in the study designs targeted by the tool, people with expertise in QA tool development and also end users, i.e. reviewers who will be using the tool. Reviewers are a group that may sometimes be overlooked but are essential to ensure that the final tool is usable by those for whom it is developed. If the tool is likely to be used in different content areas, then it is important to include reviewers who will be using the tool in all contexts. For example, ROBIS is targeted at different types of systematic reviews including reviews of interventions, diagnostic accuracy, aetiology and prognosis. We included team members who were familiar with all different types of review to ensure that the team included the appropriate expertise to develop the tool. It can also be helpful to include reviewers with a range of expertise from those new to quality assessment to more experienced reviewers. Including representatives from a wide range of organisations can also be helpful for the future uptake and dissemination of the tool. Thinking about this at an early stage is helpful. The more organisations that are involved in the development of the tool, the more likely these organisations are to feel some ownership of the tool and to want to implement the tool within their organisation in the future. The total number of people involved in tool development varies. For our tools, the number of people involved directly in the development of each tool ranged from 27 to 51 with a median of 40.

#### Manage the project

The size and the structure of the project team also need to be carefully considered. In order to cover an appropriate range of expertise, it is generally necessary to include a relatively large group of people. It may not be practical for such a large group to be involved in the day-to-day development of the tool, and so it may be desirable to have a smaller group responsible for driving the project by leading and coordinating all activities, and involving the larger group where their input is required. For example, when developing QUADAS-2 and PROBAST, a steering group of around 6–8 people led the development of the tool, bringing in a larger consensus group to help inform decisions on the scope and content of the tool. For ROBINS-I and Cochrane ROB 2.0, a smaller steering group led the development with domain-based working groups developing specific areas of the tool.

#### Define the scope

The scope of the quality assessment tool needs to be defined at an early stage. The Table 2 outlines key questions to consider when defining the scope. Tools generally target one specific type of study. The specific study design to be considered is one of the first components to define. For example, QUADAS-2 [8] focused on diagnostic accuracy studies, PROBAST [10] on prediction modelling studies and the Cochrane Risk of Bias tool on randomised trials. Some tools may be broader, targeted at multiple related designs. For example, ROBINS-I targets all non-randomised studies of interventions rather than one single study design such as cohort studies. When deciding on the focus of the tool, it is important to clearly define the design and topic areas targeted. Trade-offs of different approaches need consideration. A more focused tool can be tailored to a specific topic area. A broader tool may not be as specific but can be used to assess a wider variety of studies. For example, we developed ROBIS to be used to assess any type of systematic review, e.g. intervention, prognostic, diagnostic or aetiology. Previous tools, such as the AMSTAR tool, were developed to assess reviews of RCTs [14]. Key to any quality assessment tool is a definition of quality as addressed by the tool, i.e. defining what exactly the tool is trying to address. We have found that once the definition of quality has been clearly agreed, then it becomes much easier to decide on which items to include in the tool.

**Table 2:** Question to consider when defining the scope

● What study designs will be targeted by the new tool?
● Will the tool consider only risk of bias (internal validity) or will it also be concerned with assessing applicability (external validity) and possibly reporting quality?
● What is the definition of quality for the tool? How is risk of bias defined? How are other components of quality defined (if included), e.g. applicability?
● What type of tool structure will be adopted, e.g. simple checklist design or a domain-based approach?
● How will quality items be rated within the tool?

Other features to consider include whether to address both internal (risk of bias) and external validity (applicability) and the structure of the tool. The original QUADAS tool used a simple checklist design and combined items on risk of bias, reporting quality and applicability. Our more recently developed tools have followed a domain-based approach with a clear focus on assessment of risk of bias. Many of these domain-based tools also include sections covering applicability/relevance. How to rate individual items included in the tool also forms part of the scope. The original QUADAS tool [7] used a simple ‘yes, no or unclear’ rating for each question. The domain-based tools such as QUADAS-2, [8] ROBIS [9] and PROBAST [10] have signalling questions which flag the potential for bias. These are generally factual questions and can be answered as ‘yes, no or no information’. Some tools include a ‘probably yes’ or ‘probably no’ response to help reviewers answer these questions when there is not sufficient information for a more definite response. The overall domain ratings then use decision ratings like ‘high, low or unclear’ risk of bias. Some tools, such as ROBINS-I [11] and the RoB 2.0 [12], include additional domain level ratings such as ‘critical, severe, moderate or low’ and ‘low, some concerns, high’. We strongly recommend that at this stage, tool developers are explicit that quality scores should not be incorporated into the tools. Numerical summary quality scores have been shown to be poor indicators of study quality, and so, alternatives to their use should be encouraged [15, 16]. When developing many of our tools, we were explicit at the scope stage that we wanted to come up an overall assessment of study quality but avoid the use of quality scores. One of the reasons for introducing the domain level structure first used with the QUADAS-2 tool was explicit to avoid users calculating quality scores by simply summing the number of items fulfilled.

Agreeing the scope of the tool may not be straightforward and can require much discussion between team members. An additional consideration is how decisions on scope will be made. Will this be by a single person, by the steering group and should some or all decisions be agreed by the larger group? The approach that we have often taken is for a smaller group (e.g. steering group) to propose the scope of the tool with the agreement reached following consultation with the larger group. Questions on the scope can often form the first discussion points at a face-to-face meeting (e.g. ROBIS [9] and QUADAS-2 [8]) or the first questions on a web-based survey (e.g. PROBAST [10]).

As with any research project, a protocol that clearly defines the scope and proposed plans for the development of the tool should be produced at an early stage of the tool development process.

### Stage 2: tool development

#### Generate initial list of items for inclusion

The starting point for a tool is an initial list of items to consider for inclusion. There are various ways in which this list can be generated. These include looking at existing tools, evidence reviews and expert knowledge. The most comprehensive way is to review the literature for potential sources of bias and to provide a systematic review summarising the evidence for the effects of these. This is the approach we took for the original QUADAS tool [7] and also the updated QUADAS-2 [8, 17, 18]. Reviewing the items included in existing tools and summarising the number of tools that included each potential item can be a useful initial step as it shows which potential items of bias have been considered as important by previous tool developers. This process was followed for the original QUADAS tool [7] and for ROBIS [9]. Examining how previous systematic reviews have incorporated quality into their results can also be helpful to provide an indication of the requirements of a QA tool. If you are updating a previous QA tool then this will often form the starting point for potential items to include in the updated tool. This was the case for QUADAS-2 [8] and the RoB 2.0 [12]. For ROBINS-I [11], domains were agreed at a consensus meeting, and then expert working groups identified potential items to include in each domain. Generating the list of items for inclusion was, therefore, based on expert consensus rather than reviewing existing evidence. This can also be a valid approach. The development of PROBAST used a combined approach of using an existing tool for a related area as the starting point (QUADAS-2), non-systematic literature reviews and expert input from both steering group members and wider PROBAST group [10].

#### Agree initial items and scope

After the initial stages of tool development which can often be performed by a smaller group, input from the larger group should be sought. Methods for gaining input from the larger group include holding a face-to-face meeting or a web-based survey. At this stage, the scope defined in step 1.5 can be brought to the larger group for further discussion and refinement. The initial list of items needs to be further refined until agreement is reached on which items should be included in an initial draft of the tool. If a face-to-face meeting is held, smaller break-out groups focussing on specific domains can be a helpful structure to the meeting. QUADAS-2, ROBIS and ROBINS-I all involved face-to-face meetings with smaller break-out groups early in the development process [8, 9, 11]. If moving straight to a web-based survey, then respondents can be asked about the scope with initial questions considering possible items to include. This approach was taken for PROBAST [10] and the original QUADAS tool [7]. For PROBAST, we also asked group members to provide supporting evidence for why items should be included in the tool [10]. Items should be turned into potential questions/signalling questions for inclusion in the tool at this relatively early stage in the development of the tool.

#### Produce first draft of tool and develop guidance

Following the face-to-face meeting or initial survey rounds, a first draft of the tool can be produced. The initial draft may be produced by a smaller group (e.g. steering group), single person, or by taking a domain-based approach with the larger group split into groups with each taking responsibility for single domains. For QUADAS-2 [8] and PROBAST [10], a single person developed the first draft which was then agreed by the steering group before moving forwards. The first draft of ROBIS was developed following the face-to-face meeting by two team members. Initial drafts of ROBINS-I [11] and the RoB 2.0 [12] were produced by teams working on single domains proposing initial versions for their domains. Drafts for each domain were then put together by the steering group to give a first draft of the tool. Once a first draft of the tool is available, it may be helpful to start producing a clear guidance document describing how to assess each of the items included in the tool. The earlier such a guide can be produced, the more opportunity there will be to pilot and refine it alongside the tool.

#### Pilot and refine

The first draft of the tool needs to go through a process of refinement until a final version that has agreement of the wider group is achieved. Consensus may be achieved in various ways. Online surveys consisting of multiple rounds until agreement on the final tool is reached are a good way of involving large numbers of experts in this process. This is the approach used for QUADAS, [7], QUADAS-2 [8], ROBIS, [9] and PROBAST [10]. If domain-based working groups were adopted for the initial development of the tool, these can also be used to finalise the tool. Members of the full group can then provide feedback on draft versions, including domains that they were not initially assigned to. This approach was used for ROBINS-I and RoB 2.0. It would also be feasible to combine such an approach with a web-based survey.

Whilst the tool is being refined, initial piloting work can be undertaken. If a guidance document has been produced, then it can be included in the piloting process. If the tool is available in different formats, for example paper-based or Access database, then these could also be made available and tested as part of the piloting. The research team may ask reviewers working on appropriate review topics to pilot the tool in their review. Alternatively, reviewers can be asked to pilot the tool on a series of sample papers and to provide feedback on their experience of using the tool. An efficient way of completing such a process is to hold a piloting event where reviewers try out the tool on a sample of papers which they can either bring with them or that are provided to them. This can be a good approach to get feedback in a timely and interactive manner. However, there are costs associated with running such an event. Asking reviewers to pilot the tool in ongoing reviews can result in delays as piloting cannot be started until the review is at the data extraction stage. Identifying reviews at an appropriate stage with reviewers willing to spend the extra time needed to pilot a new tool is not always straightforward. We held a piloting event when developing the RoB 2.0 and found this to be very efficient in providing immediate feedback on the tool. We were also able to hold a group discussion for reviewers to provide suggestions for improvements to the tool and to highlight any items that they found difficult. For previous tools, we used remote piloting which provided helpful feedback but was not as efficient as the piloting event. Ideally, any piloting process should involve reviewers with a broad range of experience ranging from those with extensive experience of conducting quality assessment of studies of a variety of designs to those relatively new to the process.

The time taken for piloting and refining the tool can vary considerably. For some tools, such as ROBIS and QUADAS-2, this process was completed in around 6–9 months. For PROBAST and ROBINS-I, the process took over 4 years.

### Stage 3: dissemination

#### Develop a publication strategy

A strategy to disseminate the tool is required. This should be discussed at the start of the project but may evolve as the tool is developed. The primary means of dissemination is usually through publication in a peer-reviewed journal. A more detailed guidance document can accompany the publication and be made available as a web appendix. Another option is to have dual publications, one reporting the tool and outlining how it was developed, and a second providing additional guidance on how to use the tool. This is sometimes known as an ‘E&E’ (explanation and elaboration) publication and is an approach adopted by many reporting guidelines [13].

#### Establish a website

Developing a website for the tool can help with dissemination. Ideally, the website should be developed before publication of the tool so that details can be included in the publication. The final version of the tool can be posted on the website together with the full guidance document. Details on who contributed to the tool development and any funding should also be acknowledged on the website. Additional resources to help reviewers use the tool can also be posted there. For example, the ROBIS (www.robis-tool.info) and QUADAS (www.quadas.org) websites both contain Microsoft Access database that reviewers can use to complete their assessment and templates to produce graphical and tabular displays. They also contain links to other relevant resources and details of training opportunities. Other resources that may be useful to include on tool websites include worked examples and translations of the tools, where available. QUADAS-2 has been translated into Italian and Japanese, and the translations of these tools can be accessed via its website. If the tool has been endorsed or recommended for use by particular organisations (e.g. Cochrane, UK National Institute for Health and Care Excellence (NICE)), then this could also be included on the website.

The website is also a helpful way to encourage comments about the tool, which can lead to its further improvement, and exchange of experiences with the tool implementation.

#### Encourage uptake of tool by leading organisations

Encouraging organisations, both national and international, to recommend the tool for use in their systematic reviews is a very effective means of making sure that, once developed, the tool is used. There are different ways this can be achieved. Involving representatives from a wide range of organisations as part of the development team may mean that they are more likely to recommend the use of the tool in their organisations. Presentations at conferences, for example the Cochrane Colloquium or Health Technology Assessment Conference, may increase knowledge of the tool within that organisation making it more likely that the tool may be recommended for use. Running workshops on the tool for organisations can help increase familiarity and usability of the tool. These can also provide helpful feedback for what to include in guidance documents and to inform future updates of the tool. For example, we have been running workshops on QUADAS and ROBIS within Cochrane for a number of years. We have also provided training to institutions such as NICE on how to use the tools. QUADAS is now recommended by both these organisations, among many others, for use in diagnostic accuracy reviews. We have also run workshops on ROBIS, PROBAST, ROBINS-I and RoB 2.0 at the annual Cochrane Colloquium. We were recently approached by the Estonian Health Insurance Fund with a request to provide training to some of their reviewers so that they could implement ROBIS within their guideline development process. We supported this by running a specific training session for them.

Ultimately, the best way to encourage tool uptake is to make sure that the tool was developed robustly and fills a gap where there is currently no existing tool or there are limitations with existing tools. Ensuring that the tool is widely disseminated also means that the tool is more likely to be used and recommended.

#### Translate tools

After the tool has been published, you may receive requests to translate the tool. Translation can help to disseminate the tool and encourage its use in a much broader range of countries. Tool translations, therefore, should be encouraged but it is important to reassure yourself that the translation has been completed appropriately. One method to do this is via back translation.

## Discussion

In this paper, we suggest a framework for developing quality assessment tools. The framework consists of three stages: (1) initial steps, (2) tool development and (3) dissemination. Each stage includes defined steps that we consider important to follow when developing a tool; there is some flexibility on how these stages may be approached. In developing this framework, we have drawn on our extensive experience of developing quality assessment tools. Despite having used different approaches to the development of each of these tools, we found that all approaches shared common features and processes. This led to the development of the framework. We recommend that anyone who would like to develop a new quality assessment tool follow the stages outlined in this paper.

When developing a new tool, you need to decide how to approach each of the proposed stages. We have given some examples of how to do this, other approaches may also be valid. Factors that may influence how you choose to approach the development of your tool include available funding, topic area, number and range of people to involve, target audience and tool complexity. For example, holding face-to-face meetings and running piloting events incur greater costs than web-based surveys or asking reviewers to pilot the tool at their own convenience. More complex tools may take longer, require additional expertise, and require more piloting and refinement.

We are not aware of any existing guidance on how to develop QA tools. Moher and colleagues have produced guidance on how to develop reporting guidelines [13]. These have been cited over 190 times, mainly by new reporting guidelines, suggesting that many reporting guideline developers have found a structured approach helpful. In the absence of guidance specifically for the development of QA tools, we also based our development of QUADAS-2 [8] and ROBIS [9] on the guidance for developing reporting guidance. Although many of the steps proposed by Moher et al. apply to the development of QA tool, there are areas where these are not directly relevant and where specific guidance on developing QA tools would be helpful.

There are a very large number of quality assessment tools available. When developing ROBIS and QUADAS, we conducted reviews of existing quality assessment tools. These identified 40 tools to assess the quality of systematic reviews [19] and 91 tools to assess the quality of diagnostic accuracy studies [20]. However, only three systematic review tools (7.5%) [19] and two diagnostic tools (2%) reported being rigorously developed [20]. The lack of a rigorous development process for most tools suggests a need for guidance on how to develop quality assessment tools. We hope that our proposed framework will increase the number of tools developed using robust methods.

The large number of quality assessment tools available makes it difficult for people working on systematic reviews to choose the most appropriate tool(s) for use in their reviews. Therefore, we are developing an initiative similar to the EQUATOR Network to improve the process of quality assessment in systematic reviews. This will be known as the LATITUDES Network (www.latitudes-network.org). LATITUDES aims to highlight and increase the use of key risk of bias assessment tools, help people to use these tools more effectively, improve incorporation of results of the risk of bias assessment into the review and to disseminate best practice in risk of bias assessment.

## Conclusions

We recommend that anyone who would like to develop a new quality assessment tool follow the stages outlined in this paper. We hope that our proposed framework will increase the number of tools developed using robust methods.
